# Promotion and Prevention Processes for Sexual and Reproductive Education in Families

**DOI:** 10.1192/j.eurpsy.2026.11939

**Published:** 2026-07-31

**Authors:** K. L. Perez Correa, R. P. Torrado Diaz, O. E. Rodriguez Vega

**Affiliations:** 1 Magdalena, Institución Universitaria del Caribe, Ciénaga; 2 Magdalena, Children Internacional Colombia; 3 Magdalena, Universidad Cooperativa de Colombia, Santa Marta, Colombia

## Abstract

**Introduction:**

Teenage pregnancy remains a public health and social concern in Colombia. The family plays a crucial role as the first socialization environment and a protective factor against risky behaviors. Nevertheless, gaps persist in sexual and reproductive education, limiting effective prevention.

**Objectives:**

To identify the promotion and prevention processes for sexual and reproductive education among adolescents aged 15–17 in Commune 5 of Santa Marta, based on the educational role of parents and caregivers.

**Methods:**

A descriptive quantitative study was conducted with a representative sample of 100 parents and caregivers of adolescents. Data were collected through a structured Likert-type questionnaire and dichotomous questions, validated by five experts in psychosocial research and public health. Descriptive statistics were used to determine the frequency and distribution of responses, emphasizing knowledge, attitudes, and preventive practices related to sexual and reproductive health.

**Results:**

The analysis revealed that 92% of participants recognized the need to create understanding and supportive family environments to prevent early pregnancy, while 91% endorsed the use of contraceptive methods and 84% highlighted the importance of preventing coercive sexual relations. However, 100% reported lacking knowledge of adolescents’ sexual and reproductive rights, demonstrating the absence of a rights-based approach in family education. The results underscore a discrepancy between favorable attitudes toward prevention and the limited conceptual understanding of comprehensive sexual education within families.

**Table 1.** Table 1. long description.

Indicator	Item Evaluated	% Yes	% No
Health Approach (WHO)	Understanding and support to prevent pregnancy < 20 years	92%	8%
Healthy Sexual Promotion	Contraceptive use promotion	91%	9%
Sexual Coercion Prevention	Reduction of forced sexual relations	84%	16%
Knowledge of Rights	Awareness of sexual and reproductive rights	0%	100%

**Conclusions:**

The findings reveal a critical need to strengthen family-based sexual education programs that go beyond prevention toward empowerment and rights awareness. While parents demonstrate willingness to address sexual and reproductive issues, their lack of knowledge about adolescents’ rights limits effective prevention and open communication. It is recommended to incorporate gender-equity and human-rights perspectives in educational strategies, promoting intergenerational dialogue and collaboration between families, schools, and community institutions to improve sexual health outcomes among adolescents.

**Disclosure of Interest:**

None Declared

## Long descriptions

### Table 1. Long description

The table consists of four columns: Indicator, Item Evaluated, percent Yes, and percent No.

* Health Approach (W H O): Item evaluated is Understanding and support to prevent pregnancy less than 20 years. Results are 92 percent Yes and 8 percent No.

* Healthy Sexual Promotion: Item evaluated is Contraceptive use promotion. Results are 91 percent Yes and 9 percent No.

* Sexual Coercion Prevention: Item evaluated is Reduction of forced sexual relations. Results are 84 percent Yes and 16 percent No.

* Knowledge of Rights: Item evaluated is Awareness of sexual and reproductive rights. Results are 0 percent Yes and 100 percent No.

Navigate back to Table 1..

